# Age-related trajectories of quality of life in community dwelling older adults: findings from the Survey of Health, Aging and Retirement in Europe (SHARE)

**DOI:** 10.3389/fnagi.2025.1632607

**Published:** 2025-08-20

**Authors:** Sarah Mendorf, Konstantin G. Heimrich, Hannah M. Mühlhammer, Tino Prell, Aline Schönenberg

**Affiliations:** ^1^Department of Geriatrics, University Hospital Jena, Jena, Germany; ^2^Department of Neurology, University Hospital Jena, Jena, Germany; ^3^Department of Geriatrics, University Hospital Halle, Halle, Germany

**Keywords:** quality of life, older adults, age factors, longitudinal studies, easySHARE, SHARE

## Abstract

**Introduction:**

Previous longitudinal studies have identified numerous factors influencing quality of life (QoL) in people of older age (PoA). However, most of these studies focus on group-level trends and fail to consider individual QoL trajectories or age-specific patterns over time.

**Methods:**

We investigated longitudinal changes in QoL among community-dwelling older adults using five waves (2010–2019) of the Survey of Health, Aging and Retirement in Europe (easySHARE). Clinically relevant changes were defined via the minimal clinically important difference (MCID). We applied linear regression and linear mixed models (LMM) to explore predictors of QoL trajectories.

**Results:**

Descriptive analyses showed that 2481 PoA (19.7%) experienced stable QoL between waves, based on changes below the MCID threshold of 3.18 points. The remaining participants exhibited consistent improvements or declines, with 1,701 different longitudinal patterns of QoL identified across the five time points. These individual patterns were further examined using LMM. LMM showed that the random effect of ID had the strongest impact on QoL across the five waves, suggesting highly individual QoL patterns. The influence of age was less significant compared to ID and decreased significantly after the addition of covariates.

**Conclusion:**

Our findings underscore the importance of individual-level analyses in aging research. While QoL may appear stable at the group level, individual trajectories vary considerably. This has important implications for the use of QoL as a primary endpoint in clinical trials, particularly in geriatric populations. Notably, age alone did not significantly influence QoL over time.

## 1 Introduction

As the global population ages, understanding the longitudinal changes in quality of life (QoL) among people of older age (PoA) is becoming increasingly important. This interest is driven by the significant rise in life expectancy, which rose from 66.8 years in 2000 to 73.1 years in 2019 (WHO, 2023). These demographic shifts emphasize the importance of ensuring a longer lifespan is accompanied by high QoL despite age-related health challenges (Bowling et al., 2003).

The older population is highly diverse, and the definition of “old age” varies considerably depending on the context (Balducci, 2003). For example, while the World Health Organization typically defines PoA as individuals aged 65 and over, population studies conducted in lower-income countries often consider individuals aged 50 and over to be elderly due to differences in socioeconomic status and life expectancy (United Nations Population Fund, 2012). Furthermore, improvements in population health mean that individuals in later life today may be more functionally capable than previous generations (Dattani et al., 2023). Therefore, chronological age alone may be insufficient for capturing the complexity of aging. Gerontological research increasingly emphasizes that QoL in later life is shaped by a dynamic interplay of biological, psychological and social factors (Jeste et al., 2010). Recognizing this complexity is essential for developing frameworks that better reflect individual diversity and the multiple influences that shape aging trajectories.

In recent years, the COVID-19 pandemic has highlighted how various policies and circumstances can affect the QoL globally and individually over time (Hansel et al., 2022). While this global stressor caused a reduction in the mental and physical health of many people, not everyone was affected equally. Depending on intrinsic and extrinsic factors, some people maintained a high QoL (Malini et al., 2022). The findings prompt the question of whether QoL can be regarded as a stable, state-like construct or whether individuals are consistently confronted with changing circumstances that cause their QoL to fluctuate.

Quality of life is a multidimensional and subjective construct that refers to individuals’ perception of their physical, emotional, and social wellbeing (Dickerson et al., 2011). It is not solely determined by health but includes various life domains such as social relationships, living conditions, and autonomy (Higgs et al., 2003). Notably, some individuals may report high QoL even in the presence of physical decline, underscoring its subjective and complex nature. Given the multidimensional nature of QoL, it is unsurprising that various factors have been identified as influential for QoL in PoA. These include age, sex, body mass index (BMI), education, limitations in activities of daily living (ADL), depression/depressive symptoms, number of chronic diseases, mobility limitations, memory problems, marital status, general health, job situation, and physical activity (Awick et al., 2017; Beltz et al., 2022; Ghenţa et al., 2022; Lee et al., 2020; Makovski et al., 2019; Malhi and Mann, 2018; Noguchi et al., 2021; Payne et al., 2018; Pinzón-Rondón et al., 2022; Sivertsen et al., 2015).

Although QoL tends to decline in very old age, its relationship with age is non-linear, with periods of relative stability or even improvement in older age (Ward et al., 2019). This pattern lends support to the idea that aging is a heterogeneous process shaped by losses and compensatory adaptations (Kornadt et al., 2020).

Previous studies have examined QoL among PoA in general populations (Henchoz et al., 2019; Ward et al., 2019; Ydstebø et al., 2018) as well as special chronic diseases (Shamshirgaran et al., 2020; Yoo et al., 2016). While most studies have found only small (Henchoz et al., 2019; Ward et al., 2019), or non-significant (Ydstebø et al., 2018) average changes over time, various individual, social and health-related factors, such as cohabitation, functional limitations, mental wellbeing, loneliness and socio-economic conditions, have emerged as relevant predictors of QoL trajectories. These findings highlight the importance of moving beyond cross-sectional snapshots and developing a more nuanced and time-sensitive understanding of QoL in older age.

Yet, most existing research evaluates QoL at the group level, which can potentially mask meaningful individual variations. Further investigation is therefore needed to explore both the average trajectory and the diversity of QoL developments among PoA.

In this context, the present study aims to fill critical gaps in the literature by leveraging 9 years of longitudinal data from the Survey of Health, Aging and Retirement in Europe (SHARE) to investigate both the impact of covariates on group-level and on age-related individual levels. We also assess the influence of age, depressive symptoms, functional limitations, and other covariates on QoL changes, contributing nuanced insights relevant to both research and clinical practice.

## 2 Materials and methods

We used a freely available, longitudinal dataset on community dwelling older adults to analyze the individual change in QoL. To assess the impact of the individual against a backdrop of covariates, we selected factors already described in the literature that influence QoL in PoA: age (Pinzón-Rondón et al., 2022), sex (Lee et al., 2020), BMI (Payne et al., 2018), education (Pinzón-Rondón et al., 2022), limitations in ADL (Beltz et al., 2022), depression/depressive symptoms (Malhi and Mann, 2018; Sivertsen et al., 2015), number of chronic diseases (Makovski et al., 2019), mobility limitations (Ghenţa et al., 2022), memory problems (Ghenţa et al., 2022), marital status (Pinzón-Rondón et al., 2022), general health (Ghenţa et al., 2022; Pinzón-Rondón et al., 2022), job situation (Noguchi et al., 2021), and physical activity (Awick et al., 2017).

### 2.1 Study design and population

This research employed data from the SHARE. This is a large multinational panel dataset, comprising over 140,000 participants aged 50 and above from 20 European nations and Israel. SHARE is a research partnership that investigates the impact of health, social, economic, and environmental policies on the lives of middle-aged and elderly individuals across Europe. Currently, eight waves of SHARE data have been collected from both new and returning participants to enable cross-sectional and longitudinal data analysis of a wide range of variables. Participants were required to attend designated study centers to undergo various health assessments, such as cognitive ability tests, grip strength tests, walking speed tests, and blood tests (SHARE, 2023). During the sampling process development stage, the Country Team and Field Agency collaborate with SHARE Central to create a customized sampling design. This includes selecting a sampling frame and defining the sampling procedure. Secondly, the sample is selected by the Country Team/Field Agency or a third party, such as an institution hosting a national register. Then, it is processed to produce a gross sample file. In the third stage, SHARE Central verifies that the gross sample conforms to SHARE standards. In the final step, the gross sample data is collated and uploaded by SHARE Central and CentERdata using Sample Control software. This data is then merged with the corresponding addresses provided by the Country Team. The survey methods, sampling design, and data resources are described in detail in the respective survey materials (Börsch-Supan et al., 2013a; Börsch-Supan et al., 2013b; Börsch-Supan et al., 2015; Malter and Börsch-Supan, 2013; Malter and Börsch-Supan, 2015; Malter and Börsch-Supan, 2017). The probabilistic sampling ensured the participants chosen were nationally representative. The questionnaire was administered through a combination of computer-assisted personal interviews and a paper-pencil format. Survey questions concerned demographics, socio-relational factors, as well as health-related indices, encompassing measures of functional ability and mental health. Our study utilized data from waves 4 (2010) to 8 (2019) (Börsch-Supan, 2022a; Börsch-Supan, 2022b; Börsch-Supan, 2022c; Börsch-Supan, 2022d; Börsch-Supan, 2022e) of the simplified easySHARE dataset for analysis. Although the main release of SHARE is stored in over 100 individual data files, easySHARE conveniently stores information for all respondents and all presently released data collection waves in a single dataset. Although easySHARE is primarily intended for training purposes, it retains the full longitudinal structure and includes all the core variables that are relevant to the research questions. This approach enables the efficient and transparent modeling of change over time without compromising data integrity.

The cleaning rules for the SHARE longitudinal sample are as follows: Households in which none of the eligible members have participated in three or more consecutive waves are removed from the longitudinal sample. Non-participation may be due to non-contact, refusal, an unknown address, or any other justified reason. SHARE generally identifies and engages respondents (panelists) for interviews and has achieved a high level of success, with over 80% of respondents being panelists (Bergmann et al., 2019).

The study’s inclusion criteria required a complete measure of QoL score without any missing data. No additional exclusion criteria were specified due to the careful sampling procedure employed in SHARE and the exploratory nature of our analysis.

### 2.2 Measures

For ease of comprehension and replication, this study presents the variable names as provided in the easySHARE data set in italics (Börsch-Supan, 2022a; Börsch-Supan, 2022b; Börsch-Supan, 2022c; Börsch-Supan, 2022d; Börsch-Supan, 2022e).

#### 2.2.1 Dependent variable

The QoL is assessed by the CASP-12 score (*casp*). Four sub-dimensions - control, autonomy, self-realization, and pleasure - determine the score. These four subdomains gave the score its name. This measure distinguishes itself from health-related measures by emphasizing the positive aspects of quality of life and operating independently from external factors that could potentially impact it (Hyde et al., 2003). Control refers to one’s ability to shape their environment, while autonomy refers to the accompanying need for self-determination. Both are essential for full participation in society (Hyde et al., 2003). The domains of self-realization and pleasure pertain to the level of fulfillment of human potential and hedonic wellbeing, respectively (Higgs et al., 2003). The survey focuses on the QoL of elderly individuals (Hyde et al., 2003)

The CASP score is determined by adding up the four sub-dimensions, resulting in a scale of 12–48 points. A high score suggests higher QoL (Hyde et al., 2003). The Cronbach’s alpha values for the domains of control, autonomy, pleasure, and self-realization are 0.73, 0.33, 0.74, and 0.85, respectively (Borrat-Besson et al., 2015).

A complete assessment was available for 47,063 participants during Wave 4, compared to 54,554 who were assessed during Wave 5, 57,134 during Wave 6, 12,693 during Wave 7, and 35,277 during Wave 8.

#### 2.2.2 Independent variables

From each of the five selected waves, we derived the subsequent variables.

#### 2.2.3 Demographic variables

- Sex (female = 1, male = 0) (*female*)
- Age at interview in years (*age).* Age was divided into five groups based on the following age ranges: 50–59, 60–69, 70–79, 80–89, and 90–99 years.
- BMI in kg/m^2^ (*bmi)*.
- Marital status (*mar_stat*) with the following options: none, married and living together with spouse, registered partnership, married and living separated from spouse, never married, divorced, and widowed.
- Current job situation (*ep005_*): “In general, how would you describe your current situation?” with the options: retired, (self-) employed (including working for family business), unemployed, permanently sick or disabled, and homemaker.
- Education (*eduyears_mod*) was assessed through inquiry into their duration of full-time schooling and vocational training measured in years.

#### 2.2.4 Health-related variables

- Depressive symptomology was measured using the Center for EURO-D (eurod), which consists of 12 binary items related to symptoms such as sadness, pessimism, thoughts of suicide, guilt, sleep disturbances, reduced interest in activities, irritability, decreased appetite, fatigue, impaired concentration, lack of enjoyment, and tearfulness. Each item is assigned a score of either 0 or 1, with 1 signifying consistently a negative emotional state (i.e., 1 = higher degree of depressive symptoms). All item scores are then aggregated to derive a total score ranging from 0 to 12 (Prince et al., 1999).
- Self-rated health (SRH) (sphus) encompasses the self-assessment of health as excellent, very good, good, fair or poor
- Number of chronic diseases (chronic_mod). The index indicates how many of the following illnesses are present in each individual: myocardial infarction, hypertension, hypercholesterolemia, stroke or cerebrovascular disease, hyperglycemia or diabetes, chronic pulmonary disease, malignant neoplasm or cancer, gastric or duodenal ulcer, peptic ulcer, Parkinson’s disease, cataracts, femoral or hip fracture, other fractures, and Alzheimer’s disease.

#### 2.2.5 Functional ability

- Mobility limitations (mobilityind) were assessed by having participants indicate which activities they experienced difficulty with. The number of activities with difficulties was then tallied. The mobility evaluation included walking 100 m, walking across a room, climbing several flights of stairs, and climbing one flight of stairs. The higher the index, the greater the number of difficulties and the lower the participant’s mobility. The index scale ranges from 0 to 4.
- Vigorous activities (br015_) provides information on the frequency of engaging in vigorous activities such as sports, physically demanding jobs, and strenuous household chores. Participants were asked to choose from the options of ‘more than once a week,” “once a week,” “one to three times a month,” and “hardly ever or never.”
- Limitations of ADL (adla) were evaluated by asking participants to mark the activities they have difficulties with. The sum was calculated, encompassing the following ADLs: dressing, including putting on shoes and socks; bathing or showering; eating, such as cutting up your food; walking across a room; and getting in or out of bed. The higher the index is the more difficulties with these activities and the lower the mobility of the respondent. adla ranges from 0 to 5.

#### 2.2.6 Memory problems to evaluate cognitive function

- The first recall trial (recall_1) assesses the number of correctly remembered words with scores ranging from 0 to 10. This was employed to assess cognition (Mansbach et al., 2014).

### 2.3 Statistical analysis

All statistical analyses were performed utilizing IBM SPSS statistics (Version 29) and R (Version 4.1.1). The included parameters did not conform to a normal distribution, as confirmed by the Shapiro-Wilk test. Therefore, metric values are presented as median with interquartile range (IQR). Nominal and ordinal values are reported as numbers and percentages. Missing data were handled through pairwise deletion. The Little’s test of missing completely at random test (Little et al., 1995) was not significant (χ^2^ = 633,787, df = 56,123, *p* = 0.07), missing completely at random can therefore be assumed and bias is not an issue. All statistical tests were conducted in a two-tailed manner with a level of significance set at 0.05. First, we compared all variables for PoA with complete CASP in all five waves in terms of their change within waves. To compare the five waves, we conducted a Friedman test for ordinal and metric variables and Cochrane’s Q for nominal variables with post hoc analysis adjusted according to Bonferroni correction.

Our next objective was to evaluate the influence of variables on QoL development across various waves. Firstly, we intended to identify when a shift in the CASP score across waves could be viewed as a meaningful change. Considering the wide range of possible CASP scores, utilizing any change starting with a single point difference is not feasible due to measurement errors and memory bias. Instead, we chose the minimal clinically important difference (MCID) as the clinically significant difference, taking into consideration the standard deviation (SD) of CASP scores. We calculated this using the distribution method: MCID = 0.5 * SD (Norman et al., 2004).

In our case, the formula was:


$$\mathrm{MCID}=0.5^{*}[({\mathrm{SD}}_{\mathrm{wave4}}+{\mathrm{SD}}_{\mathrm{wave5}}+{\mathrm{SD}}_{\mathrm{wave6}}+{\mathrm{SD}}_{\mathrm{wave7}}+$$

$${\mathrm{SD}}_{\mathrm{wave8}})/5]=0.5{}^{*}[(6.44+6.25+6.34+$$

6.47+6.26)/5]= 3.18

The MCID of 3.18 was used to identify clinically significant CASP-12 differences between waves of at least four points. Participants were then divided into two groups based on the MCID: those with stable QoL across all five waves, and those with fluctuating QoL across the waves. Stable QoL denotes no more than a three-point difference in participants’ CASP scores between the waves, while unstable QoL indicates a difference of at least four points between at least two waves. To investigate potential disparities between individuals with stable and unstable QoL, we performed a group comparison using the U-test and Chi^2^-test.

However, it should be noted that the definition of the MCID specifically incorporates this information: It has been defined as the smallest score difference in the relevant score that patients perceive as advantageous and that would demand a change in the patient’s treatment while ensuring there are no severe side effects or exorbitant costs (Jaeschke et al., 1989). Osoba et al. (1998) highlight the significance of identifying clinically meaningful differences, since even minor numerical disparities in mean health-related QoL scores may yield statistically significant results with generous sample sizes, but statistical significance is not interchangeable with clinical significance.

One advantage of distribution-based methods is their ability to account for changes beyond a certain level of random variation (Crosby et al., 2003). Distribution-based methods are specific to the sample used. For instance, statistical analysis alone can extract MCID scores from a study with a large sample size and wide distribution, even if no actual change has occurred (Wright et al., 2012).

However, distribution-based methods have a weakness in that there are few established benchmarks for determining clinically significant improvements. More significantly, distribution-based methods are inadequate in addressing the question of a patient’s perspective of clinically important change, which varies distinctly from statistical significance (Crosby et al., 2003).

In order to assess the differing patterns in QoL, we computed the CASP sum score differences for each participant across Wave 4 to Wave 8, including Wave 4–Wave 5, Wave 4–Wave 6, Wave 4–Wave 7, Wave 4–Wave 8, Wave 5–Wave 6, Wave 5–Wave 7, Wave 5–Wave 8, Wave 6–Wave 7, Wave 6–Wave 8, and Wave 7–Wave 8. Differences of ± 4 or more were classified as ± 1, indicating a clinically significant increase or decrease between the respective waves. Differences below 4 were coded as 0. We subsequently analyzed the number of unique patterns of increase, decrease and stability observed in the longitudinal subsample.

Finally, to identify the factors that contribute to changes in QoL, a linear mixed model (LMM) was used to investigate the correlation between CASP scores and (1) fixed intercept with only the waves, and (2) with the inclusion of covariates such as waves, gender, current job status, limitations in ADL, educational level in years, BMI, marital status, number of chronic diseases, SRH, EURO-D, mobility limitations, physical activities, and word recall as fixed effect predictors. ID and age were implemented as the fixed effect predictor, while random intercepts for participants were specified to account for the repeated measures nature of the data. The model’s fit was evaluated using Akaike’s information Criterion (AIC), and the fixed effects’ significance was determined based on p-values. Furthermore, we calculated the Intraclass Correlation Coefficient (ICC) using the given formula:


$$\frac{v\,a\,r\,i\,a\,n\,c\,e\,o\,f\,r\,a\,n\,d\,o\,m\,e\,f\,f\,e\,c\,t}{\left(v\,a\,r\,i\,a\,n\,c\,e\,o\,f\,r\,a\,n\,d\,o\,m\,e\,f\,f\,e\,c\,t+v\,a\,r\,i\,a\,n\,c\,e\,o\,f\,r\,e\,s\,i\,d\,u\,a\,l\right)}$$

To validate the appropriateness of LMM, we checked model assumptions via visual inspection of residual plots and Q-Q plots. No substantial violations were found: residuals were approximately normally distributed, and homoscedasticity was largely met. Thus, no data transformations were required.

To account for individual differences in change over time, we estimated random slopes for the time variable (wave) at the participant level. This allowed the rate of change to vary between individuals. Models with random slopes [specified as (wave | id)] were compared to random intercept-only models using AIC, R^2^, and residual diagnostics to evaluate potential improvements in model fit.

Linear mixed models enable adequate modeling of the dependencies between measurement time points and allow the simultaneous estimation of fixed effects (e.g., time, covariates) and random effects (e.g., individual differences between participants) (Singer and Willett, 2003). This allows individual changes in QoL to be differentiated over time.

## 3 Results

The sociodemographic data for the participants included in this study can be found in Supplementary Table 1. The median CASP score remained stable across the entire cohort over time, with a range of 38–39. The specific changes in all factors between the waves are visualized in Supplementary Figure 1, while the corresponding statistical information can be found in Supplementary Tables 1, 2.

### 3.1 Development of QoL

To analyze QoL trajectories, we included a subsample of 12,603 participants who had provided complete CASP data across all five waves. The baseline characteristics of this cohort, as assessed at Wave 4, are presented in Table 1. We utilized a MCID of ≥ 4 points to determine a significant difference in CASP score between waves. In total, 80.3% (*n* = 10,122) demonstrated significant changes in QoL across all five waves (unstable QoL). When examining the underlying structures responsible for these CASP alterations, 1,701 patterns were identified. These patterns illustrate alterations (either an increase, a decrease or no change) between waves 4–5, waves 4–6, waves 4–7, waves 4–8, waves 5–6, waves 5–7, waves 5–8, waves 6–7, waves 6–8, and waves 7–8 that surpass the identified MCID. For this purpose, we descriptively compared the CASP values between all available wave pairs at group level.

**TABLE 1:** Comparison between stable and unstable quality of life (QoL).

Wave 8		Total *n* = 12,603	Stable QoL *n* = 2,481 (19.7%)	Unstable QoL *n* = 10,122 (80.3%)	U-Test with R^2^
		**Median (IQR)**	**Median (IQR)**	**Median (IQR)**	
Age in years		70 (70–80)	70 (70–80)	70 (70–80)	0.003***
Education in years		12 (8–14)	12 (8–15)	11 (8–14)	0.008***
Number of chronic diseases		1 (0–2)	1 (0–2)	1 (0–2)	0.003***
CASP		39 (35–43)	42 (39–45)	38 (34–42)	0.062***
BMI in kg/m^2^		26 (24–29)	26 (23–29)	27 (24–30)	0.004***
EURO-D		2 (1–4)	1 (0–3)	2 (1–4)	0.020***
Recall of words		5 (4–7)	6 (5–7)	5 (4–6)	0.014***
Limitations of ADL		0 (0)	0 (0)	0 (0)	0.006***
Mobility limitations		0 (0)	0 (0)	0 (0–1)	0.013***
		***n* (%)**	***n* (%)**	***n* (%)**	**Chi^2^-test with Cramers V**
Sex	Male	5,090 (40.4%)	1,028 (41.4%)	4,062 (40.1%)	*P* = 0.235
	Female	7,513 (59.6%)	1,453 (58.6%)	6,060 (59.9%)	
Marital status	Married and living together with spouse	7,807 (62.0%)	1,649 (66.5%)	6,158 (60.9%)	0.060***
	Registered partnership	162 (1.3%)	32 (1.3%)	130 (1.3%)	
	Married, living separated from spouse	142 (1.1%)	21 (0.8%)	121 (1.2%)	
	Never married	734 (5.8%)	144 (5.8%)	590 (5.8%)	
	Divorced	1,276 (10.1%)	260 (10.5%)	1,016 (10.0%)	
	Widowed	2,473 (19.6%)	373 (15.0%)	2,100 (20.8%)	
SRH	Excellent	745 (5.9%)	270 (10.9%)	475 (4.7%)	0.189***
	Very good	2,240 (17.8%)	674 (27.2%)	1,566 (15.5%)	
	Good	4,884 (38.8%)	947 (38.2%)	3,937 (38.9%)	
	Fair	3,686 (29.2%)	492 (19.8%)	3,194 (31.6%)	
	Poor	1,047 (8.3%)	97 (3.9%)	950 (9.4%)	
Vigorous activities	More than once a week	3,832 (30.4%)	975 (39.3%)	2,857 (28.2%)	0.115***
	Once a week	1,804 (14.3%)	385 (15.5%)	1,419 (14.0%)	
	One to three times a month	1,154 (9.2%)	249 (10.0%)	905 (8.9%)	
	Hardly ever, or never	5,806 (46.1%)	870 (35.1%)	4,936 (48.8%)	
Current job situation	Retired	9,700 (77.8%)	1,914 (77.6%)	7,786 (77.9%)	0.060***
	(Self-)employed	1,625 (13.0%)	390 (15.8%)	1,235 (12.4%)	
	Unemployed	117 (0.9%)	15 (0.6%)	102 (1.0%)	
	Permanently sick or disabled	191 (1.5%)	18 (0.7%)	173 (1.7%)	
	Homemaker	699 (5.6%)	106 (4.3%)	593 (5.9%)	
	Other	130 (1.0%)	22 (0.9%)	108 (1.1%)	

Effect sizes are denoted in bold and indicated as statistically significant by asterisks ***p < 0.001. IQR, interquartile range; BMI, body mass index; ADL, activities of daily living; CASP, QOL questionnaire; EURO-D, depressive symptoms questionnaire; SRH, self-rated health.

Group comparisons revealed that individuals with unstable QoL had higher age, lower education, a higher number of chronic illnesses, increased limitations in ADLs, lower scores on the CASP questionnaires, higher BMI, more depressive symptoms, greater mobility limitations, decreased ability to recall words, poor SRH, were more frequently widowed, less frequently (self-) employed, more frequently unemployed and sick, and were less inclined towards vigorous activities. Importantly, the effect sizes were small (Supplementary Table 2).

### 3.2 Linear mixed models

To gain a comprehensive understanding of the varying CASP scores over different periods, we conducted LMMs. Initially, we distinguished between group and individual effects, as diverse patterns indicate a highly diverse QoL trajectory across the five waves. Subsequently, we carried out an initial simple model (Table 2), consisting of the corresponding wave’s CASP score as the dependent variable, alongside wave as a fixed and ID and age intervals as a random effect. The analysis reveals that the null hypothesis that CASP remains the same between waves has been refuted by the ANOVA, F(4, 205,636) = 219.1, *p* < 0.001, meaning that CASP differs across the waves. Further inspection of the descriptive results indicates that this variation primarily stems from the difference between all waves and wave five (*p* < 0.001) (Supplementary Figure 1 and Supplementary Table 1). By evaluating the simple regression model (Table 3) with model slope and intercept, these findings are reinforced:

**TABLE 2:** Linear mixed models (LMM) with waves.

	CASP: quality of life and wellbeing index		
Predictors	Estimates	CI	*P*
(Intercept)	35.85	34.15–37.55	**< 0.001**
Wave 5	0.49	0.44–0.54	**< 0.001**
Wave 6	0.22	0.16–0.27	**< 0.001**
Wave 7	0.34	0.25–0.43	**< 0.001**
Wave 8	0.67	0.60–0.73	**< 0.001**
**Random effects**	**Value**		
σ^2^	13.78		
τ_00__*id*_	25.16		
τ_00__*age_intervals*_	3.76		
ICC	0.68		
N _*id*_	92,723		
N _*age_intervals*_	5		
Observations	205,641		
Marginal R^2^/conditional R^2^	0.001/0.678		

CASP, QOL questionnaire, CI, confidence interval, ICC, intraclass correlation coefficient, LMM, linear mixed model. Significant values are marked in bold.

**TABLE 3:** Simple linear regression between CASP and waves.

	CASP: quality of life and wellbeing index		
Predictors	Estimates	CI	*P*
(Intercept)	37.17	37.11–37.22	**< 0.001**
Wave 5	0.95	0.87–1.03	**< 0.001**
Wave 6	0.04	−0.03–0.12	0.278
Wave 7	−0.07	−0.20– 0.05	0.266
Wave 8	0.56	0.47– 0.65	**< 0.001**
Observations	205,641		
R^2^/R^2^ adjusted	0.004/0.004		

CASP, QoL questionnaire; CI, cofidence interval. Significant values are marked in bold


$$f\,\left(x\right)=37.17\,-\,0.95\,{}^{*}\,\,{\mathrm{QoL}}_{\mathrm{wave}\,\,5}-\,0.04\,{}^{*}\,\,{\mathrm{QoL}}_{\mathrm{wave6}}-$$

$$\left(-0.07\right)\,{}^{*}\,\,{\mathrm{QoL}}_{\mathrm{wave7}}-\,0.56\,{}^{*}\,\,{\mathrm{QoL}}_{\mathrm{wave8}}$$

This indicates that QoL increased by an average of 0.95 points between wave 4 and wave 5, by 0.04 points between wave 4 and wave 6, decreased by 0.07 between wave 4 and 7, and increased by 0.56 points between wave 4 and 8. These findings suggest that on a group level, QoL remains relatively stable in line with the descriptive results presented in Table 1.

However, the random effect revealed a significant impact of ID and age on the CASP. The analysis of the explained variance reveals a variance of 25.16 for the random effect of ID and 3.76 for the random effect of age intervals, with only 13.78 residual variance. The calculated ICC indicates a high level of intra-personal clustering at 0.68, showing that 68% of the model variance can be explained by ID and age alone (Table 3).

**FIGURE 1:**
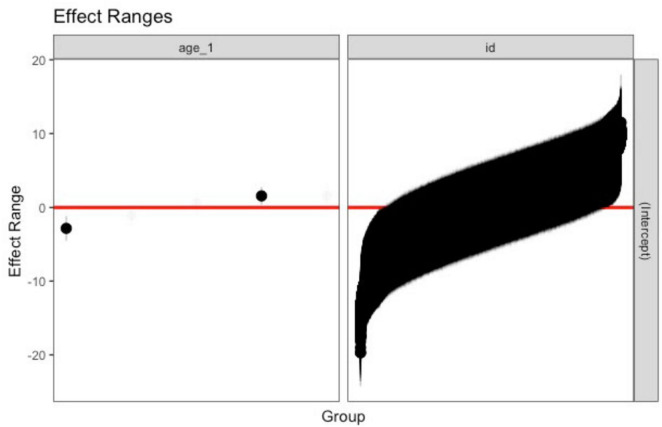
Estimated random effects (intercept, estimated random effects; age_1, age).

Figure 1 displays the estimated random effects (intercept) for each ID and age (*age_1*) along with their confidence interval estimation. Bold intervals do not include zero and demonstrate IDs that are starting out relatively higher or lower compared to a “typical” ID. These results exhibit a wide range of random effects, indicating that CASP scores vary on an individual level between waves.

**FIGURE 2:**
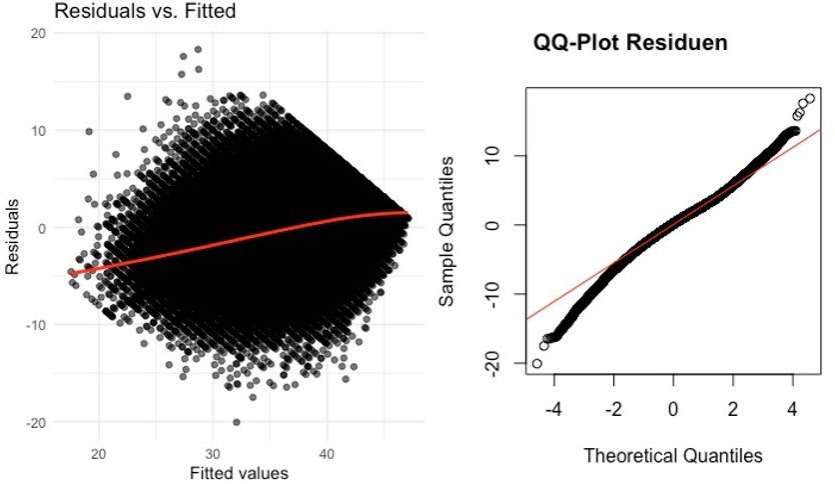
Residual plots and Q-Q plots for linear mixes model (LMM) with waves.

**FIGURE 3:**
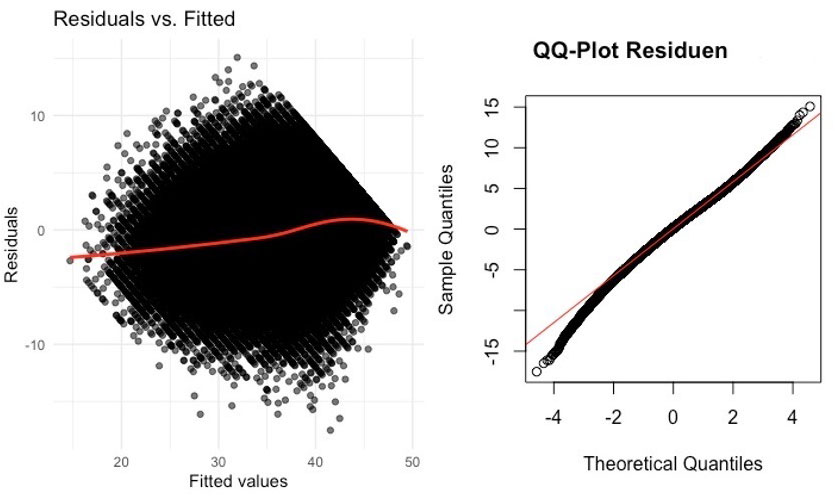
Residual plots and Q-Q plots for linear mixes model (LMM) with covariates.

To assess the impact of ID and age relative to the previously identified covariates, we conducted a second linear mixed model, with CASP as the independent variable and various covariates as fixed effects (Table 4). We checked model assumptions via visual inspection of residual plots and Q-Q plots (Figures 2, 3). Residuals were approximately normally distributed, and homoscedasticity was largely met. ANOVA analysis for model comparison showed that the model, comprising covariates, had a better fit compared to the initial model (*p* < 0.001), demonstrating the additional covariates contribute toward comprehending the alterations in CASP. Examining the covariates revealed a noteworthy negative impact on QoL for depressive symptoms (EURO-D), education level, number of chronic diseases, SRH, BMI, recall of words, limitations in mobility and ADL, physical intensity of activities, present job situation, marital status, and gender. Identifying the random effect of ID and age, an ICC of 0.48 emerged, and the conditional R^2^ was notably higher than the marginal R^2^, indicating a powerful influence of ID and age on the CASP scores. However, the random effect of age was greatly reduced compared to ID, falling to only 0.05. Meanwhile, the random effect of ID only decreased to 11.5. Marginal R^2^ shows the variance explained solely by fixed factors, while conditional R^2^ takes into account the variance explained by both fixed and random effects.

**TABLE 4:** Linear mixed models (LMM) with covariates.

	CASP: quality of life and wellbeing index		
Predictors	Estimates	CI	*P*
(Intercept)	37.76	37.49–38.04	**< 0.001**
Wave 5	0.31	0.27–0.36	**< 0.001**
Wave 6	−0.04	−0.09–0.01	0.118
Wave 7	0.05	−0.04–0.13	0.271
Wave 8	0.30	0.24–0.36	**< 0.001**
Sex – female	0.40	0.34–0.46	**< 0.001**
Years of education	0.11	0.10–0.11	**< 0.001**
Marital status – registered partnership	0.17	−0.04–0.38	0.121
Marital status – never married	−0.61	−0.73 to −0.48	**< 0.001**
Marital status - divorced	−0.61	−0.71 to −0.51	**< 0.001**
Marital status – widowed	−0.23	−0.30 to −0.15	**< 0.001**
SRH	−3.29	−3.38 to −3.21	**< 0.001**
Number of chronic diseases	−0.10	−0.12 to −0.08	**< 0.001**
EURO-D	−0.87	−0.88 to −0.86	**< 0.001**
Limitations ins ADL	−0.18	−0.22 to −0.14	**< 0.001**
Mobility limitations	−0.79	−0.82 to −0.76	**< 0.001**
BMI	−0.01	−0.02 to −0.01	**< 0.001**
Vigorous activities	−0.51	−0.55 to −0.47	**< 0.001**
Current job situation – (self-)employed	0.03	−0.04–0.10	0.403
Current job situation – unemployed	−1.41	−1.54 to −1.27	**< 0.001**
Current job situation – permanently sick	−0.52	−0.65 to −0.39	**< 0.001**
Current job situation – homemaker	−0.90	−0.99 to −0.81	**< 0.001**
Current job situation – other	−0.48	−0.65 to −0.31	**< 0.001**
Recall of words	0.22	0.21–0.24	**< 0.001**
**Random effects**	**Value**		
σ^2^	12.42		
τ_00__*id*_	11.50		
τ_00__*age_intervals*_	0.05		
ICC	0.48		
N _*id*_	92,723		
N _*age_intervals*_	5		
Observations	205,641		
Marginal R^2^/conditional R^2^	0.346/0.661		

CASP, QOL questionnaire; SRH, self-rated health; EURO-D, depressive symptoms questionnaire; ADL, activities of daily living; BMI, body mass index; CI, confidence interval, ICC, intraclass correlation coefficient; LMM, linear mixed model. Significant values are marked in bold.

Additionally, we explored models with random slopes for the time variable, which allowed individual trajectories to vary. Therefore, wave modeled as a continuous variable with random slopes at the individual level (Supplementary Tables 3, 4 and Supplementary Figures 2, 3). A precise interpretation can be found in the appendix under the corresponding tables. Although these models showed meaningful slope variance (τ*11* = 0.36–0.59) and a theoretically plausible negative intercept–slope correlation (ρ = −0.66 to −0.75), they did not substantially improve overall model fit (as measured by AIC or conditional R^2^) and showed only minimal gains in residual structure. Therefore, we retained the models without random slopes as the most parsimonious and substantively appropriate solution for our analyses and added the analysis with random slopes in the supplement.

## 4 Discussion

Our study highlights a critical insight into the nature of QoL trajectories among older adults: while average group-level analyses indicate relative stability over nearly a decade, individual-level data reveal a rich tapestry of dynamic changes. Over 80% of participants experienced clinically meaningful fluctuations in QoL, illustrating that aging is a highly individualized process influenced by complex biopsychosocial factors.

### 4.1 Age as a limited predictor of QoL change

Chronological age is often used as a proxy for functional decline and reduced well-being. However, our findings - consistent with previous research – show that age itself explains little variance in QoL once other factors are taken into account (Ferrucci et al., 2020).

While age does influence QoL, this effect is smaller than that of individual factors and becomes negligible once additional covariates are considered. This reflects the significant individual differences in the aging process (Kornadt et al., 2020).

These results highlight that biological age, psychosocial context, and comorbidities play a more crucial role in shaping QoL. They also challenge age-based policies and categorizations, advocating for more nuanced, functionally oriented approaches (Sanderson and Scherbov, 2008). In this regard, we found that individual factors have a substantial impact on QoL. Clinical assessments of aging should, therefore, take into account the diversity of older adults, including life expectancy, disease prevalence, functional dependence, cognitive abilities, emotional health, and socio-economic resources (Stuck et al., 1993).

### 4.2 Individual variability and the limitations of group averages

Our research revealed that the QoL for PoA appears stable when assessed using traditional group comparison or regression methods. However, when examining recent changes in QoL, we found that only 19.7% of PoA experienced stable QoL based on a MCID of 4. The study uncovered 1,701 distinct QoL trajectories, highlighting the limitations of conventional group-level analyses, which often obscure meaningful intra-individual variations (Zaninotto et al., 2009). This variability likely results from the interaction of multiple personal, environmental, and contextual factors, such as health events, social support, psychological resilience, and life transitions (Ong et al., 2009). Our findings reinforce the need for more personalized approaches in gerontological research, moving beyond averages to better capture the diverse individual experiences of aging.

This conclusion aligns with other studies showing that PoA maintain a relatively stable QoL, with only slight, non-significant declines [e.g., (Gobbens and van Assen, 2014; Ydstebø et al., 2018)]. These findings support the idea that PoA represent a highly diverse group (Balducci, 2003), which is further reflected in the different QoL patterns observed. The growing proportion of PoA in the population due to demographic shifts underscores the importance of understanding their QoL development. It is crucial to note that the number of identified patterns depends on the number of time points assessed - in this case, five. Researchers may not always be aware of the specific phase of the trajectory when evaluating QoL, which can affect interpretations, especially in longitudinal data collection (Singer and Willett, 2003).

While QoL is often considered stable in group analyses, our findings suggest significant fluctuations in QoL during the study period, without altering median values. This fluctuation poses a challenge for using QoL as a single endpoint in clinical trials, as it is difficult to determine the exact phase of the QoL trajectory when measurements are taken. To address this, we used mathematical analysis to identify patterns and avoid masking individual influences by grouping data into general categories. Additionally, we employed MCID to assess significant QoL changes, rather than relying solely on raw QoL scores. Our analysis also suggests that factors such as age, memory problems, and functional capabilities have a considerable impact on shaping these QoL trajectories. Another potential reason for the lack of marked changes in QoL could be the larger and more diverse participant group in our study, which included a broader range of ages and cognitive and functional limitations than in previous studies (Henchoz et al., 2019; Ward et al., 2019; Ydstebø et al., 2018).

### 4.3 Methodological implications

One of the key strengths of this study is the use of the MCID to distinguish meaningful changes in QoL from statistical noise, providing clinically interpretable insights that are often lacking in large epidemiological studies (Norman et al., 2003). It has been defined as the smallest score difference in the relevant score that patients perceive as advantageous and that would demand a change in the patient’s treatment while ensuring there are no severe side effects or exorbitant costs (Jaeschke et al., 1989). Osoba et al. (1998) highlight the significance of identifying clinically meaningful differences, since even minor numerical disparities in mean health-related QoL scores may yield statistically significant results with generous sample sizes, but statistical significance is not interchangeable with clinical significance. The significant individual fluctuations observed suggest that cross-sectional or single time-point assessments may fail to capture the complexity of QoL changes, potentially leading to misleading conclusions about intervention outcomes (Seves et al., 2021).

Our LMM results reveal that individual QoL developments tend to balance out at the group level. While some individuals experience an improvement in QoL, others face a decline, which ultimately cancels out at the aggregate level. However, considerable variability at the individual level underscores the importance of personal circumstances - rather than medical, social, or psychological factors alone - in shaping QoL among PoA. This reinforces the idea that QoL in PoA is highly individualized and diverse. These findings are further supported by a study in which QoL in PoA did not necessarily correlate with declines in clinical parameters, and patients’ self-reported QoL scores were higher than those rated by proxies (Ydstebø et al., 2018).

Our primary research aim was to identify age-related trajectories of QoL. For this purpose, models that treat wave as a categorical fixed effect (without random slopes) provide clearer, more interpretable estimates and better address our research questions. These models also showed higher marginal R^2^, indicating better explanatory power of the fixed predictors. Therefore, the models without random slopes were retained as the most parsimonious and substantively appropriate solution for our analyses.

### 4.4 Psychosocial and functional determinants

However, we have identified further variables that account for a decrease in QoL, in addition to individual trajectories.

Depressive symptoms are a powerful predictor of QoL in PoA, a finding that aligns with extensive research demonstrating the profound impact of mental health on subjective wellbeing in later life. They also shown a consistent and significant association between the severity of depression and lower QoL, a relationship that remains stable across various QoL evaluation instruments over time (Sivertsen et al., 2015). This reinforces the critical role of mental health in shaping QoL trajectories.

The connection between depressive symptoms and QoL of PoA is in link with previous research. A review demonstrated a noteworthy link between the severity of depression and lower QoL in PoA. Furthermore, this association remained consistent over time, regardless of which QoL evaluation instruments were utilized (Sivertsen et al., 2015).

In addition to depressive symptoms, other factors such as functional limitations and SRH also play significant roles in QoL development. Limitations in ADL and education have been shown to influence QoL in PoA, as highlighted by Henchoz et al. (2019). The interdependence of physical health and psychological wellbeing is further underscored by the fact that SRH is a well-established factor influencing QoL (Brückner et al., 2024) and functional limitations directly affect both physical and mental health (Gobbens and van Assen, 2014).

Cognitive function, although a weaker predictor, still contributes to QoL, particularly in terms of autonomy and social engagement (Gamage et al., 2018). These findings collectively suggest that while mental health and physical limitations are central to QoL in PoA, cognitive function also plays an important supporting role.

However, the literature on gender differences in QoL remains inconclusive, with mixed findings across studies (Cramm and Nieboer, 2015; Gale et al., 2014; Gobbens and van Assen, 2014). This indicates the need for further exploration into how gender interacts with other factors to shape QoL in later life.

Muscle Resistance and Walking Ability (with one query for each mobility limitations) as well as Illness were predictive factors in our study, allowing us to demonstrate that factors associated with frailty have a detrimental effect on QoL. Frailty is identified as a specific clinical syndrome when meeting three or more out of the five phenotypic criteria using the Fried criteria (Buta et al., 2016). Furthermore, multimorbidity is evaluated based on eleven predetermined diseases (Chen et al., 2014). The limitations of surveying frailty using the Fried criteria have led to the development of a simplified questionnaire instrument that asks about comparable criteria – the FRAIL-Scale (Benzinger et al., 2021):

- Fatigue - Do you feel tired most of the time?
- Muscle Resistance - Can you climb one floor of stairs?
- Walking Ability - Can you walk 100 m?
- Illness - Do you suffer from more than five illnesses?
- Weight Loss - Have you unintentionally lost more than 5 kg in the last 6 months?

Hu et al. (2023) reported the bidirectional relationship between frailty and QoL. A high initial level of frailty predicted poorer QoL in the future (β = −0.151, *p* < 0.001), as did initial low QoL predicting increases in frailty later on (β = −0.052, *p* < 0.001). Furthermore, early changes in frailty were found to predict later changes in QoL. Chronic illnesses have been also shown to have a particularly negative impact on the physical aspect of the wellbeing of PoA (Maresova et al., 2019).

In general, it is suggested that future research considers individual differences in QoL over time to accurately assess wellbeing and intervention effectiveness. Along with these idiosyncratic factors, including medical, social, and psychological factors, also affect QoL trajectories and should be addressed in interventions. Furthermore, it has been demonstrated that the impact of age on its own is minimal and is also influenced by individual factors. To determine the age, it is important to consider the diverse characteristics of the older population, including life expectancy, disease incidence and prevalence, functional dependence, cognition, emotions, and socio-economic resources.

Our results resonate with theories of successful and healthy aging emphasizing adaptability, resilience, and person-environment fit (Trica et al., 2024). The dynamic nature of QoL trajectories suggests that interventions enhancing resilience and coping resources could mitigate declines and foster sustained wellbeing. On a societal level, these findings highlight the importance of social determinants of health, such as social support, socioeconomic status, and access to healthcare, which modulate QoL trajectories and aging outcomes (Marmot, 2005). Aging policies should thus address these broader determinants to promote equitable health and QoL in aging populations. These findings have practical relevance for healthcare providers and policymakers. Interventions targeting depressive symptoms, promoting physical activity, and supporting cognitive health could help maintain or improve QoL. Importantly, the heterogeneity observed calls for tailored approaches that consider individual risk and protective factors rather than “one-size-fits-all” solutions.

## 5 Limitations

Several variables in the analysis depend on self-reports, including QoL, depressive symptoms, SRH, and ADL, indicating that the data provided cannot be objectified. Self-reported data presents challenges, including the inclination of individuals to provide socially desirable responses and the possibility of sampling bias (Devaux and Sassi, 2015). However, it has been suggested by research that self-reports possess crucial information (Lenderink et al., 2012) and all self-report measures used in data collection are validated and widely applied. Furthermore, the use of self-report measures is essential in comprehending a subjective construct like QoL, which cannot be objectively assessed. In addition, SHARE has implemented a standardized, computer-assisted personal interviewing data collection method to reduce the impact of response bias. Furthermore, personal evaluations prevail over objective measurements regarding the QoL.

Despite its advantages, such as large sample sizes and standardized data collection procedures, the use of a multi-national panel dataset may introduce bias toward the inclusion of comparably healthy participants. However, SHARE counteracted this risk by utilizing purposive sampling to include a representative sample. Furthermore, computer-assisted interviewing was used to guide participants through the questionnaires and reduce missing data. While it cannot be ruled out that missing data may be more prevalent in participants with poorer mental and physical health, a Little’s test confirmed that the missing in the current dataset are consistent with a completely random mechanism. This reduces concerns regarding systematic attrition or survivorship bias.

Additionally, it is necessary to determine a significant cut-off point for evaluating the clinical relevance of changes in QoL scores. Improvement of a mere one point on a questionnaire with up to 48 points cannot genuinely reflect a meaningful change in QoL. We reject the idea of utilizing any change in the QoL score, as this would lead to complex patterns that are tough to interpret. Therefore, we employed the MCID, which considers the distribution of QoL scores in the analyzed sample. Nevertheless, alternative approaches warrant investigation, accounting for individual differences. It is noteworthy that scores exceeding the 4-point MCID cut-off were not distinguished for ease of interpretation. However, in future studies, evaluating the distinction between minor and significant QoL changes could be advantageous, though defining these changes may require further consideration. In future studies, to gain a deeper understanding of the point at which patients perceive a subjectively meaningful change in their QoL, analyses could be supplemented with detailed qualitative data.

The heterogeneity of the QoL trajectories present challenges in terms of prediction and generalizability of the findings. Nevertheless, this heterogeneity highlights the complexity of QoL development in older adults. Our findings do not seek to replace cross-sectional assessments; rather, they demonstrate that single time-point measures may overlook significant longitudinal patterns. The results should emphasize the value of longitudinal approaches and the variability hidden by group averages.

The stability of QoL is assessed using the CASP, which already takes into account the specific characteristics of QoL in relation to age. To gain a better understanding of QoL in PoA, it may be beneficial to repeat the analyses using disease-specific questionnaires.

## 6 Conclusion

This study provides compelling evidence that QoL in older age is not a static or uniformly declining construct, but a highly dynamic and individualized experience. Despite apparent stability at the population level, over 80% of individuals in our cohort experienced clinically relevant changes in QoL over a 9 years period. This divergence between group-level trends and individual trajectories underscores the need to shift away from age-based generalizations and toward more nuanced, person-centered approaches in aging research and care. Our findings show that chronological age alone is a poor predictor of QoL change. Instead, psychosocial and functional variables - particularly depressive symptoms, perceived health, mobility limitations, and cognitive status - demonstrated stronger associations with QoL outcomes. These factors are, in many cases, modifiable, suggesting opportunities for targeted interventions to improve wellbeing in later life.

From a methodological standpoint, our study highlights the limitations of single time-point assessments and emphasizes the value of longitudinal, individualized measures. The use of the MCID allowed us to capture meaningful change beyond statistical variation, providing a more accurate reflection of older adults’ lived experiences. These insights carry important implications for public health policy, clinical decision-making, and the design of future research. Interventions to support QoL should prioritize mental health, functional autonomy, and social engagement, rather than relying solely on chronological indicators. Future studies should also explore resilience mechanisms and subjective perceptions of aging to better understand why some individuals maintain high QoL despite adversity. QoL remains a central but complex indicator of aging. A nuanced understanding of its individual trajectories will be critical for designing responsive healthcare systems and targeted public health policies for aging populations.

## Data Availability

Publicly available datasets were analyzed in this study. This data can be found here: http://www.share-project.org.
