# Supplementary material for: Age-related trajectories of quality of life in community dwelling older adults: findings from the Survey of Health, Aging and Retirement in Europe (SHARE)
Source: Front Aging Neurosci. 2025 Aug 20;17:1632607. doi: 10.3389/fnagi.2025.1632607 (PMC12405344; doi:10.3389/fnagi.2025.1632607)
Supplement: Supplementary file 1 [file Table_1.docx]

Suppl. Table 1. Demographic characteristics and comparison between the waves for PoA

| Variables | | **wave 4**  **(**n = 47,063) | **wave 5**  **(**n = 54,554) | **wave 6**  **(**n = 57,134) | **wave 7**  **(**n = 11,613) | **wave 8**  **(**n = 35,277) | **wave comparison** |
| --- | --- | --- | --- | --- | --- | --- | --- |
|  |  | **median (IQR)** | **median (IQR)** | **median (IQR)** | **median (IQR)** | **median (IQR)** |  |
| Age in years | | 65 (58-73) | 65 (59-73) | 66 (60-74) | 71 (66-78) | 70 (64-77) | **<0.001** |
| BMI in kg/m^2^ | | 26 (24-29) | 26 (24-29) | 26 (24-29) | 26 (24-29) | 27 (24-30) | **<0.001** |
| Education in years | | 11 (8-13) | 11 (8-14) | 11 (8-14) | 11 (8-14) | 12 (9-14) | 1.000 |
| limitations in ADL | | 0 (0) | 0 (0) | 0 (0) | 0 (0) | 0 (0) | **<0.001** |
| CASP | | 38 (33-42) | 39 (34-43) | 38 (33-42) | 38 (33-42) | 38 (34-43) | **<0.001** |
| EURO-D | | 2 (1-4) | 2 (1-4) | 2 (1-4) | 2 (1-4) | 2 (1-4) | **<0.001** |
| number of chronic diseases | | 1 (0-2) | 1 (0-2) | 1 (0-2) | 1 (0-2) | 1 (0-2) | **<0.001** |
| Mobility limitations | | 0 (0-1) | 0 (0-1) | 0 (0-1) | 0 (0-1) | 0 (0-1) | **<0.001** |
| Recall of words | | 5 (4-6) | 5 (4-7) | 5 (4-7) | 5 (4-6) | 5 (4-7) | **<0.001** |
|  |  | **n (%)** | **n (%)** | **n (%)** | **n (%)** | **n (%)** | **wave comparison** |
| Sex | Male | 20884 (44.4%) | 24580 (45.1%) | 25329 (44.3%) | 5008 (43.1%) | 15115 (42.8%) | 1.000 |
|  | Female | 26179 (55.6%) | 29974 (54.9%) | 31805 (55.7%) | 6605 (56.9%) | 20162 (57.2%) |  |
| Marital status | Married | 32696 (69.5%) | 38701 (70.9%) | 40155 (70.3%) | 7964 (68.6%) | 23946 (67.9%) | **<0.001** |
|  | Registered partnership | 742 (1.6%) | 818 (1.5%) | 801 (1.4%) | 113 (1.0%) | 529 (1.5%) |  |
|  | Never married | 2537 (5.4%) | 2971 (5.4%) | 3091 (5.4%) | 570 (4.9%) | 1768 (5.0%) |  |
|  | Divorced | 4271 (9.1%) | 4943 (9.1%) | 4964 (8.7%) | 817 (7.0%) | 3036 (8.6%) |  |
|  | Widowed | 6817 (14.5%) | 7121 (13.1%) | 8123 (14.2%) | 2149 (18.5%) | 5998 (17.0%) |  |
| SRH | Excellent | 3291 (7.0%) | 4771 (8.7%) | 4117 (7.2%) | 655 (5.6%) | 2181 (6.2%) | **<0.001** |
|  | Very good | 7837 (16.7%) | 9985 (18.3%) | 10684 (18.7%) | 2158 (18.6%) | 6275 (17.8%) |  |
|  | Good | 16657 (35.4%) | 20342 (37.3%) | 20977 (36.7%) | 4583 (39.5%) | 13860 (39.3%) |  |
|  | Fair | 13808 (29.3%) | 14542 (26.7%) | 16047 (28.1%) | 3243 (27.9%) | 10063 (28.5%) |  |
|  | Poor | 5470 (11.6%) | 4914 (9.0%) | 5309 (9.3%) | 974 (8.4%) | 2898 (8.2%) |  |
| Current job situation | Retired | 27054 (57.5%) | 30835 (56.5%) | 33574 (58.8%) | 8655 (74.5%) | 24441 (69.3%) | **<0.001** |
|  | (self-)employed | 12790 (27.2%) | 15683 (28.7%) | 14591 (25.5%) | 1262 (10.9%) | 6579 (18.6%) |  |
|  | Unemployed | 1556 (3.3%) | 1588 (2.9%) | 1679 (2.9%) | 106 (0.9%) | 622 (1.8%) |  |
|  | Permanently sick | 1665 (3.5%) | 1778 (3.3%) | 1550 (2.7%) | 221 (1.9%) | 796 (2.3%) |  |
|  | Homemaker | 3509 (7.5%) | 4128 (7.6%) | 4730 (8.3%) | 1137 (9.8%) | 2357 (6.7%) |  |
|  | Other | 489 (1.0%) | 542 (1.0%) | 1010 (1.8%) | 232 (2.0%) | 482 (1.4%) |  |
| Vigorous activities | more than once a week | 15828 (33.6%) | 19856 (36.4%) | 19498 (34.1%) | 3369 (29.0%) | 11331 (32.1%) | **<0.001** |
|  | once a week | 6536 (13.9%) | 7817 (14.3%) | 8404 (14.7%) | 1758 (15.1%) | 5299 (15.0%) |  |
|  | one to threee times a month | 4352 (9.2%) | 4720 (8.7%) | 5785 (10.1%) | 1334 (11.5%) | 3946 (11.2%) |  |
|  | hardly ever or never | 20347 (43.2%) | 22161 (40.6%) | 23447 (41.0%) | 5152 (44.4%) | 14701 (41.7%) |  |
| IQR = Interquartile range, BMI = body mass index, ADL = activities of daily living, CASP = QOL questionnaire, EURO-D = depressive symptoms questionnaire, SRH = self-rated health | | | | | | | |
