# Supplementary material for: Age-related trajectories of quality of life in community dwelling older adults: findings from the Survey of Health, Aging and Retirement in Europe (SHARE)
Source: Front Aging Neurosci. 2025 Aug 20;17:1632607. doi: 10.3389/fnagi.2025.1632607 (PMC12405344; doi:10.3389/fnagi.2025.1632607)
Supplement: Supplementary file 2 [file Table_2.docx]

**Suppl. Table 2. Friedman test with post hoc analysis**

| **variable** | **Friedman test** | **post hoc analysis** | | | | | | | | | |
| --- | --- | --- | --- | --- | --- | --- | --- | --- | --- | --- | --- |
|  |  | **Wave 4-5** | **Wave 4-6** | **Wave 4-7** | **Wave 4-8** | **Wave 5-6** | **Wave 5-7** | **Wave 5-8** | **Wave 6-7** | **Wave 6-8** | **Wave 7-8** |
| Age in years | **<0.001** | **<0.001** | **<0.001** | **<0.001** | **<0.001** | **<0.001** | **<0.001** | **<0.001** | **<0.001** | **<0.001** | **<0.001** |
| Education in years | 1.000 | 1.000 | 1.000 | 1.000 | 1.000 | 1.000 | 1.000 | 1.000 | 1.000 | 1.000 | 1.000 |
| Number of chronic diseases | **<0.001** | 1.000 | **<0.001** | **<0.001** | **<0.001** | **<0.001** | **<0.001** | **<0.001** | **<0.001** | **<0.001** | **<0.001** |
| CASP | **<0.001** | **<0.001** | **0.022** | 1.000 | 1.000 | **<0.001** | **<0.001** | **<0.001** | 0.151 | **0.008** | 1.000 |
| BMI in kg/m^2^ | **<0.001** | **<0.001** | **<0.001** | **<0.001** | **<0.001** | **0.001** | **0.001** | **0.003** | 1.000 | **<0.001** | **<0.001** |
| EURO-D | **<0.001** | 1.000 | 1.000 | 0.634 | **<0.001** | 1.000 | 0.197 | **<0.001** | **0.035** | **<0.001** | **<0.001** |
| Recall of words | **<0.001** | 1.000 | 0.143 | **<0.001** | **<0.001** | **1.000** | **<0.001** | **<0.001** | **<0.001** | **<0.001** | **<0.001** |
| Limitations of ADL | **<0.001** | 1.000 | 0.378 | **0.009** | **<0.001** | 1.000 | 0.158 | **<0.001** | 1.000 | **<0.001** | **<0.001** |
| SRH | **<0.001** | **0.042** | **<0.001** | **<0.001** | **<0.001** | **<0.001** | **<0.001** | **<0.001** | **<0.001** | **<0.001** | 0.165 |
| Vigorous activities | **<0.001** | 0.921 | 0.053 | **0.023** | **<0.001** | 1.000 | 1.000 | **<0.001** | 1.000 | **<0.001** | **<0.001** |
| CASP = QOL questionnaire, BMI = body mass index, EURO-D = depressive symptoms questionnaire, ADL = activities of daily living, SRH = self-rated health | | | | | | | | | | | |
