# Supplementary material for: Age-related trajectories of quality of life in community dwelling older adults: findings from the Survey of Health, Aging and Retirement in Europe (SHARE)
Source: Front Aging Neurosci. 2025 Aug 20;17:1632607. doi: 10.3389/fnagi.2025.1632607 (PMC12405344; doi:10.3389/fnagi.2025.1632607)
Supplement: Supplementary file 3 [file Table_3.docx]

**Suppl. Table 3. LMM with waves and random slopes**

|  | **CASP: quality of life and well-being index** | | |
| --- | --- | --- | --- |
| *Predictors* | *Estimates* | *CI* | *p* |
| (Intercept) | 38.23 | 38.12 – 38.34 | **<0.001** |
| wave | -0.03 | -0.05 – -0.01 | **0.001** |
| **Random Effects** | | | |
| σ^2^ | 11.89 | | |
| τ_00_ _id_ | 41.76 | | |
| τ_11_ _id.wave_ | 0.59 | | |
| ρ_01_ _id_ | -0.66 | | |
| ICC | 0.67 | | |
| N _id_ | 35115 | | |
| Observations | 124417 | | |
| Marginal R^2^ / Conditional R^2^ | 0.000 / 0.673 | | |
| CASP = QOL questionnaire, CI = Confidence interval,  ICC = Intraclass Correlation Coefficient, LMM = linear mixed model | | | |

The LMM with waves and random slopes revealed a minimal linear time trend of -0.03 points per wave. At the same time, however, there was strong inter-individual variance in both the baseline level of well-being (τ₀₀ = 41.76) and the change over time (τ₁₁ = 0.59), and a significant negative correlation was observed between the two (ρ = -0.66). This suggests that people with a higher CASP score tend to experience greater declines over time.
