# Supplementary material for: Age-related trajectories of quality of life in community dwelling older adults: findings from the Survey of Health, Aging and Retirement in Europe (SHARE)
Source: Front Aging Neurosci. 2025 Aug 20;17:1632607. doi: 10.3389/fnagi.2025.1632607 (PMC12405344; doi:10.3389/fnagi.2025.1632607)
Supplement: Supplementary file 4 [file Table_4.docx]

**Suppl. Table 4.** **LMM with covariates and random slopes**

|  | **CASP: quality of life and well-being index** | | |
| --- | --- | --- | --- |
| *Predictors* | *Estimates* | *CI* | *p* |
| (Intercept) | 39.55 | 39.08 – 40.02 | **<0.001** |
| wave | 0.11 | 0.10 – 0.13 | **<0.001** |
| Sex – female | 0.15 | 0.07 – 0.24 | **<0.001** |
| Years of education | 0.08 | 0.07 – 0.09 | **<0.001** |
| Marital status – Registered partnership | 0.32 | -0.00 – 0.64 | 0.053 |
| Marital status – Married, living separated from spouse | -0.96 | -1.29 – -0.63 | **<0.001** |
| Marital status – Never married | -0.59 | -0.77 – -0.42 | **<0.001** |
| Marital status – Divorced | -0.65 | -0.78 – -0.52 | **<0.001** |
| Marital status – Widowed | -0.14 | -0.24 – -0.03 | **0.012** |
| SRH | -3.18 | -3.28 – -3.07 | **<0.001** |
| Number of chronic diseases | -0.14 | -0.16 – -0.11 | **<0.001** |
| EURO-D | -0.78 | -0.80 – -0.77 | **<0.001** |
| Limitations ins ADL | -0.23 | -0.28 – -0.18 | **<0.001** |
| Mobility limitations | -0.72 | -0.76 – -0.68 | **<0.001** |
| BMI | -0.02 | -0.03 – -0.02 | **<0.001** |
| vigorous activities | -0.55 | -0.62 – -0.49 | **<0.001** |
| Current job situation – (self-)employed | -0.10 | -0.18 – -0.01 | **0.023** |
| Current job situation – unemployed | -1.18 | -1.36 – -1.01 | **<0.001** |
| Current job situation – permanently sick | -0.58 | -0.75 – -0.40 | **<0.001** |
| Current job situation – homemaker | -0.92 | -1.04 – -0.80 | **<0.001** |
| Current job situation – other | -0.29 | -0.51 – -0.08 | **0.007** |
| Recall of words | 0.21 | 0.19 – 0.23 | **<0.001** |
| **Random Effects** | | | |
| σ^2^ | 11.11 | | |
| τ_00_ _id_ | 24.70 | | |
| τ_11_ _id.wave_ | 0.36 | | |
| ρ_01_ _id_ | -0.75 | | |
| ICC | 0.51 | | |
| N _id_ | 35115 | | |
| Observations | 124417 | | |
| Marginal R^2^ / Conditional R^2^ | 0.300 / 0.659 | | |
| CASP = QOL questionnaire, SRH = self-rated health, EURO-D = depressive symptoms  questionnaire, ADL = activities of daily living, BMI = body mass index, CI = Confidence interval,  ICC = Intraclass Correlation Coefficient, LMM = linear mixed model | | | |

The LMM with covariates and random slopes revealed a significant linear increase in CASP values over time (β = 0.11, p < 0.001). The random-slope variance (τ₁₁ = 0.36) and the strong negative correlation between the starting level of each individual and their subsequent change (ρ = -0.75) suggest that there are substantial differences between individuals in terms of how the course progresses.
