# Supplementary material for: Age-related trajectories of quality of life in community dwelling older adults: findings from the Survey of Health, Aging and Retirement in Europe (SHARE)
Source: Front Aging Neurosci. 2025 Aug 20;17:1632607. doi: 10.3389/fnagi.2025.1632607 (PMC12405344; doi:10.3389/fnagi.2025.1632607)
Supplement: Supplementary file 5 [file Data_Sheet_1.docx]

| 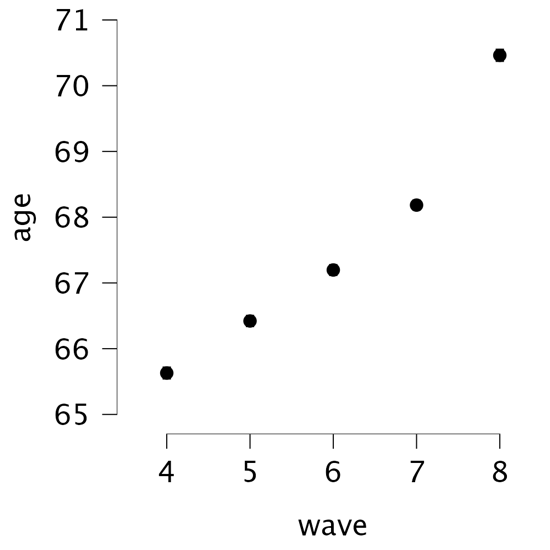 | 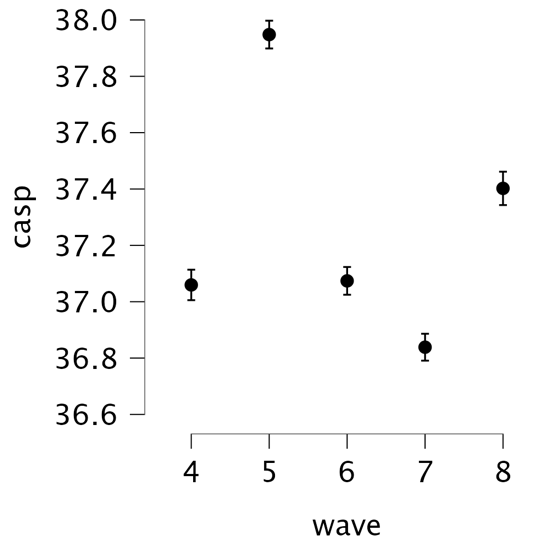 | 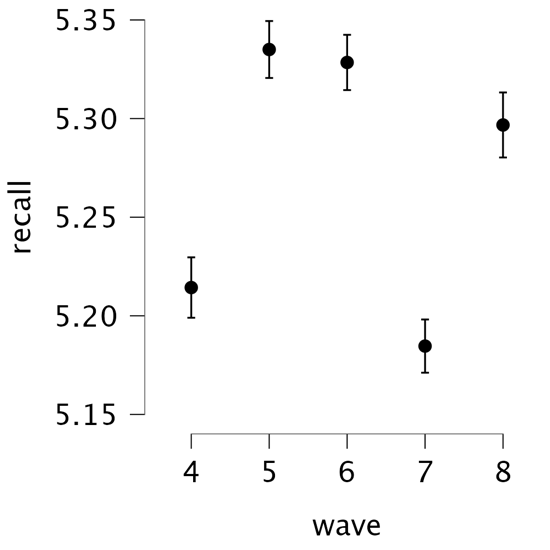 |
| --- | --- | --- |
| 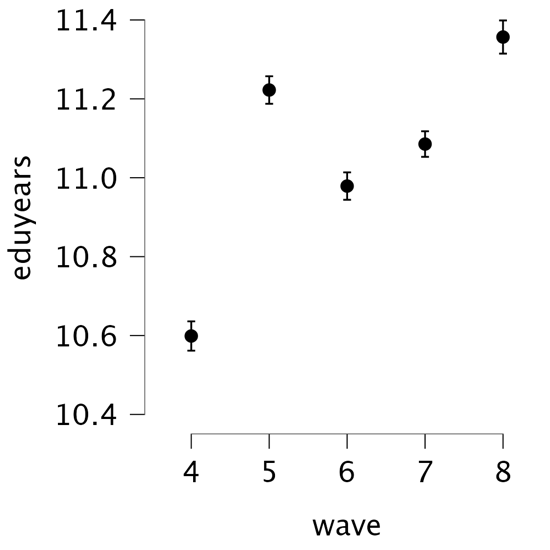 | 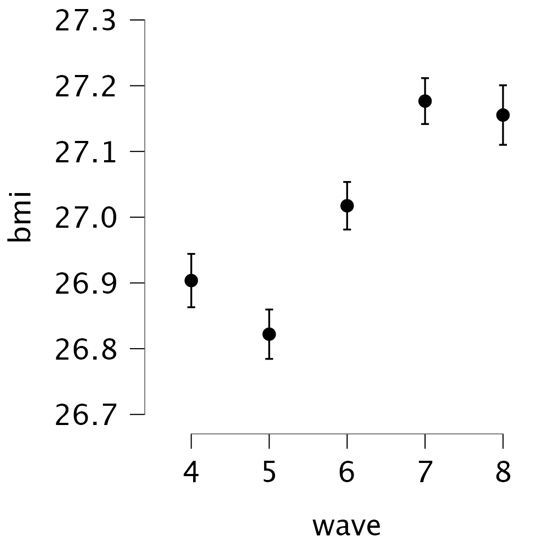 | 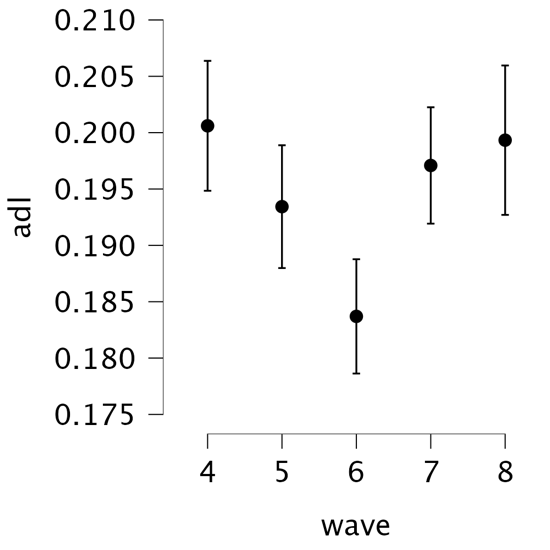 |
| 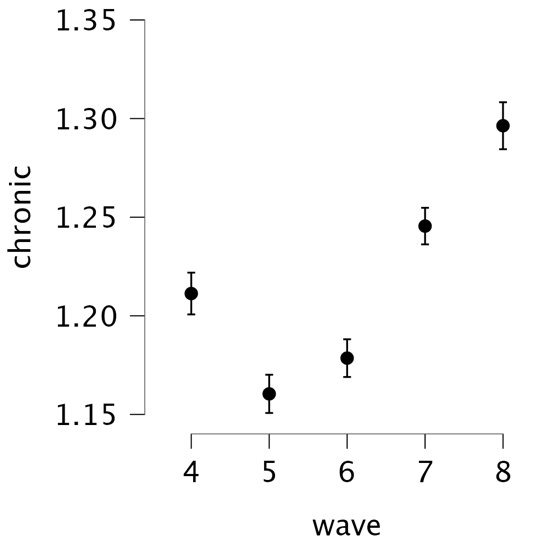 | 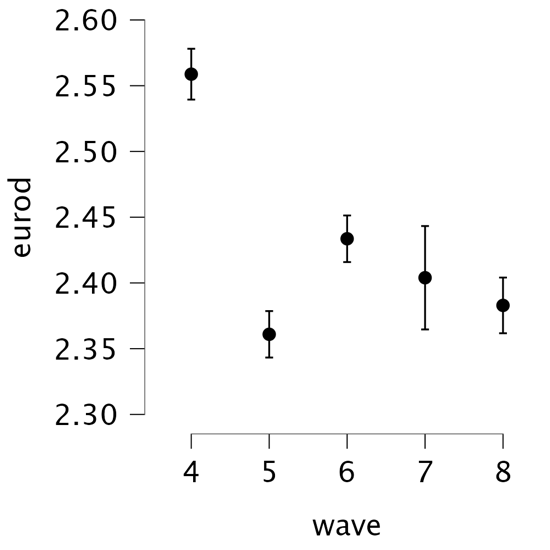 | 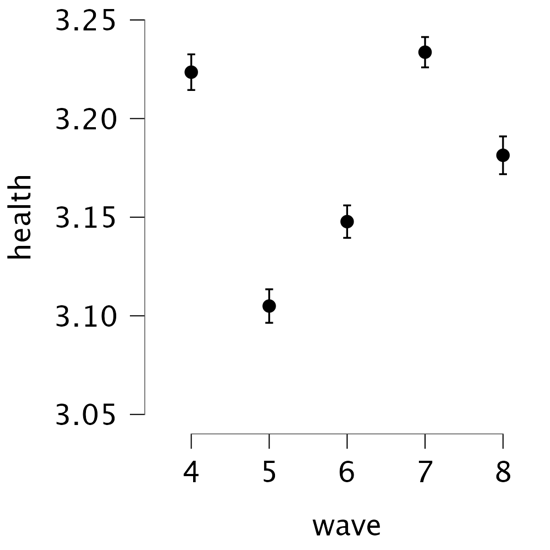 |
| Eduyears = education in years, bmi = Body mass index, adl = limitations in activites of daily living, chronic = number of chronic diseases, health = self-rated health | | |

**Suppl. Figure 1. Interval plots with mean and standard deviation**
