# Supplementary figures and images for: Age-related trajectories of quality of life in community dwelling older adults: findings from the Survey of Health, Aging and Retirement in Europe (SHARE)
Source: Front Aging Neurosci. 2025 Aug 20;17:1632607. doi: 10.3389/fnagi.2025.1632607 (PMC12405344; doi:10.3389/fnagi.2025.1632607)

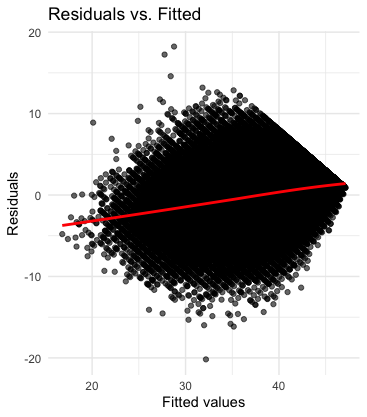

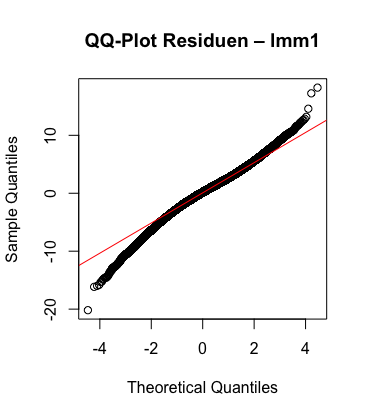


**Suppl. Figure 2. Residual and QQ-Plot for LMM with waves and random slopes**

Supplement: Supplementary file 6 [file Data_Sheet_2.docx]

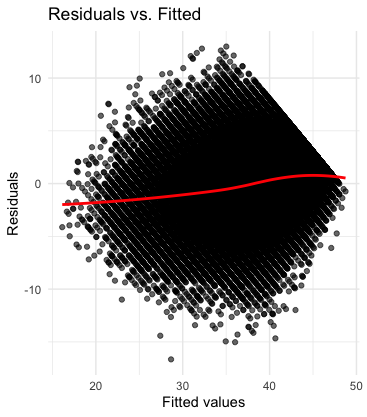

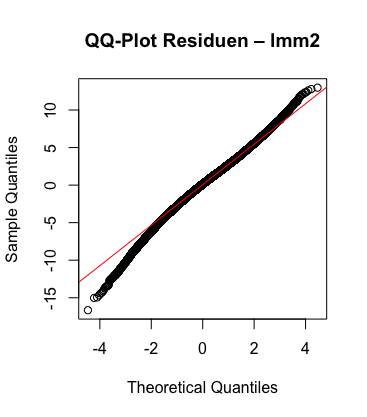


**Suppl. Figure 3. Residual and QQ-Plot for LMM with covariates and random slopes**

Supplement: Supplementary file 7 [file Data_Sheet_3.docx]
